# Identification of AAV variants with improved transduction of human vascular endothelial cells by screening AAV capsid libraries in non-human primates

**DOI:** 10.1038/s41434-025-00563-4

**Published:** 2025-08-22

**Authors:** Maria Stamataki, Julia Lüschow, Christina Schlumbohm, Malik Alawi, Lars Lunding, Eberhard Fuchs, Martin Trepel, Markus Schwaninger, Jakob Körbelin

**Affiliations:** 1 ENDomics Lab, Department of Oncology, Hematology and Bone Marrow Transplantation, University Medical Center Hamburg-Eppendorf, Hamburg, Germany; 2 German Primate Center – Leibniz-Institute for Primatology, Göttingen, Germany; 3 Bioinformatics Core, University Medical Center Hamburg-Eppendorf, Hamburg, Germany; 4 Division of Lung Immunology, Research Center Borstel, Borstel, Germany; 5 Department of Hematology and Medical Oncology, Augsburg University Hospital and Medical Faculty, Augsburg, Germany; 6 Bavarian Cancer Research Center (BZKF), Augsburg, Germany; 7 Comprehensive Cancer Center Alliance WERA, Augsburg, Germany; 8 Institute for Experimental and Clinical Pharmacology and Toxicology, University of Lübeck, Lübeck, Germany; 9 German Research Centre for Cardiovascular Research (DZHK), partner site Hamburg/Lübeck/Kiel, Lübeck, Germany

**Keywords:** Genetic vectors, Genetic vectors

## Abstract

The development of targeted vector systems for gene therapy has made impressive progress during the last decade. Promising vector candidates were identified by screening large pools of adeno-associated virus (AAV) mutants in small animal models. However, it became apparent that targeted AAV mutants isolated from rodents may not function in humans as the tropism of individual AAV mutants can differ between species. To identify novel vascular-targeted AAV capsid mutants suitable for treating human patients, we generated a set of AAV2 display peptide libraries and screened them in the common marmoset, a non-human primate. To evaluate the impact of different AAV library production methods, progress of the screening process was monitored by next generation sequencing. Particle distribution and enrichment was compared between different AAV libraries and selection rounds. We observed enrichment of AAV variants in the brain and other well-perfused organs (lung, heart, kidney) potentially mediated by high capsid affinity for the vascular endothelium in general. In vitro experiments on primary human microvascular endothelial cells isolated from a set of different organs (brain, heart, lung, liver, kidney and spleen) confirmed superior transduction of a selected AAV variant displaying the “DWP” amino acid sequence motif compared to natural AAV serotypes 1–9.

## Introduction

Due to their safety profile, the capability to transduce different cell types and easily scalable production protocols, vectors based on AAV have successfully been used in many preclinical studies, found their way into numerous clinical trials and were approved as gene therapy drugs for routine clinical use [1]. While some organs like the liver are transduced rather easily after intravenous injection of different available AAV serotypes, other organs like the brain either require more invasive methods of vector application (like intracerebral injection) or they depend on molecularly tailored AAV capsid variants optimized for the intended target tissue. Multiple attempts aimed at improving AAV’s tropism for different cell types by modifying the viral capsid. Capsid shuffling of AAV serotypes [2–4], attachment or incorporation of cell type-specific antibodies, nanobodies or other ligands like DARPins [5–7], and AAV display peptide libraries [8–11] have been employed to generate more efficient and more specific AAV vectors directed towards the cell type or organ of interest. The AAV9-based AAV.PHP.B capsid family with improved neurotropism in mice, for example, has been identified by screening AAV display peptide libraries over multiple selection rounds in vivo [12, 13]. Different laboratories including ours set focus on the brain vascular endothelium leading to the identification of AAV capsid variants such as “AAV-BR1” (AAV2 presenting the peptide NRGTEWD) [14] or AAV BI30 (AAV9 presenting the peptide NNSTRGG) [15] selectively targeting the blood-brain barrier (BBB)- associated endothelium of the cerebral microvasculature in mice. In the past years it became apparent, however, that screening AAV capsid libraries in rodents does not necessarily yield variants that are efficient in primates. One intriguing example is the above-mentioned AAV9 mutant AAV.PHP-B. Showing strong tropism for the brain and the ability to cross the BBB after intravenous injection in C57BL/6 mice, this AAV variant clearly outperforms wildtype AAV9 and other brain-directed vectors. It’s exceptional brain tropism, however, is not only restricted to rodents but even limited to certain mouse strains, due to a polymorphism in its target receptor LY6a in C57BL/6 mice [16–18]. Similar species-restrictions likely apply to other AAV capsid mutants, also to AAV-BR1 [19]. Being interested in brain endothelial-targeted AAV capsids for clinical applications, we therefore decided to repeat the in vivo screening of our AAV display peptide libraries in an animal model more closely related to humans. As model we chose the common marmoset (*Callithrix jacchus*), a small non-human primate which has gained attraction as “star of Japan’s ambitious brain project” [20]. The BBB of marmosets and humans show close similarity [21, 22] and due to the small body size, the dose of AAV library particles injected in these animals is suitable for AAV capsid screenings. Further, marmoset monkeys have recently been used to successfully screen for brain-directed AAV variants [23]. We chose peptide libraries based on AAV2 as this serotype had been shown to yield almost completely liver-detargeted variants upon peptide insertion [14, 24], whereas most so-far described AAV9 mutants still confer profound homing to the liver and other peripheral organs after intravenous injection if not modified additionally [12, 13, 15, 25, 26]. As insertion site we chose amino acid position R588 (VP1 numbering) which had previously been used successfully in mice in multiple settings [14, 24, 27, 28]. We introduced peptides of different lengths and compared different AAV library production methods in order to enhance diversity of the particle pool. In some of the applied AAV libraries we excluded the amino acid cysteine as this amino acid had previously been shown to be highly underrepresented in AAV-displayed peptides potentially interfering with capsid assembly and/or stability [29, 30]. The screening process was monitored by quantitative assessment of particle numbers in the brain and multiple peripheral organs after each selection round followed by next generation sequencing (NGS) of isolated capsid mutants from the respective organs. Peptide sequences were scored for enrichment and organ-specificity and clustered into consensus motifs. Performing a total of six selection rounds, we identified AAV capsid mutants with increased homing to the marmoset brain and other well perfused organs (lung, heart, kidney) potentially being targeted to vascular endothelial cells (EC) in general. On primary human microvascular endothelial cells from various organs, increased EC transduction was confirmed in vitro compared to natural AAV serotypes 1–9.

## Materials & methods

### Preparation of a random AAV display peptide libraries

In this study we used random AAV display peptide libraries comprising AAV2 particles with different modifications at nucleotide position 3967 in the AAV genome (corresponding to amino acid position R588; VP1 numbering).AAV library particles either displayed six amino acids (encoded as X6 fixed trimers not including a cysteine codon), seven random amino acids (encoded by the degenerated NNK scheme including a cysteine codon or as X7 fixed trimers not including a cysteine codon), or twelve amino acids (encoded as X12 fixed trimers not including a cysteine codon). The libraries each contained about 2 × 10^8^ unique clones and were produced either in two steps via AAV transfer shuttles, analogous to previously described protocols [9, 31] or by direct transfection in one step as described before [31]. In short, the oligonucleotides encoding the randomized peptides were synthesized (NNK_7_: Metabion, Martinsried, Germany; Trimer_6/7/12_-C: Ella Biotech, Fürstenfeldbruck, Germany) according to following scheme: 5’-CAGTCGGCCAGAGAGGC(random_insert)GCCCAGGCGGCTGACGAG-3’. The second strand was synthesized using the sequenase kit (Thermo Fisher) and the starting primer 5’-CTCGTCAGCCGCCTGG-3’. The double-stranded insert was cleaved with *Bgl*I, purified with the QIAquick Nucleotide Removal Kit (Qiagen, Hilden, Germany) and ligated into the *Sfi*I-digested pMT187-0-3 library plasmid [9]. The diversity of the plasmid library was determined by the number of clones growing from a representative aliquot of transformed electro competent DH5αbacteria on agar containing 150 mg/mL ampicillin. Library plasmids were harvested and purified using the Nucleobond Maxi kit (Macherey& Nagel). HEK 293 T cells were grown in DMEM (Gibco) containing 10% FCS (Gibco) at 37°C with 5% CO_2_. The “one-step” libraries were produced by directly transfecting 5 × 10^8^ 293T-cells in a total of fifty 15 cm cell culture dishes (1 × 10^7^ cells per dish) with 100 ng plasmid library and 11.9 µg pXX6 adenoviral helper plasmid [32] per dish (corresponding to 500 plasmid library copies per cell) using Polyfect transfection reagent (Qiagen). In case of the “two-steps” libraries, the AAV library genomes were packaged into chimeric AAV library capsids containing AAV2 wildtype VP subunits (AAV transfer shuttles) by transfecting 5 × 10^8^ 293T-cells in a total fifty 15 cm cell culture dishes (1 × 10^7^ cells per dish) at a 1:1:2 ratio of the plasmid pVP3cm (containing the wildtype cap gene with modified codon usage, without inverted terminal repeats) [33], the library plasmids and the pXX6 helper plasmid [32]. The resulting AAV library transfer shuttles were used to infect a total of 5 × 10^8^ 293 T cells in fifty 15 cm cell culture dishes (1 × 10^7^ cells per dish) at a multiplicity of infection (MOI) of 0.1 replicative units per cell. Cells were superinfected with Ad5 at an MOI of 5 plaque-forming units (pfu)/cell.

The final random peptide AAV display libraries were harvested from the cell culture supernatant after 48 h (two-steps) or 72 h (one-step). The supernatant was precipitated by PEG8000/NaCl [34] and purified by iodixanol density gradient ultracentrifugation [35]. The purified iodixanol-purified AAV library was re-purified by HighTrap AVB sepharose (GE Healthcare) according to manufacturer’s instruction and dialyzed against HBSS. The virus titer was determined from 1:10,000 diluted samples at genomic level by real-time PCR.

### Determination of AAV copy numbers by qPCR

To determine viral copy numbers, we used the SYBR Green I-based FastStart Essential DNA Green Master (Roche) with the Light Cycler Nano system (Roche). An initial denaturation of the probes (95 °C, 10 min) was followed by 45 cycles of amplification (95 °C/67 °C/72 °C; 30 sec each; rampage 5 °C/sec) and a final melting curve analysis (60 °C to 97 °C with 0.1 °C/sec). For each vector, corresponding plasmid DNA was used to generate a standard curve. *Cap*-specific primers were used for titration of AAV libraries: Cap_fw: 5′-GCAGTATGGTTCTGTATCTACCAACC-3′ and Cap_rev: 5′-GCCTGGAAGAACGCCTTGTGTG-3’, whereas promoter-specific primers were used for titration of recombinant AAV vectors: CAG_fw: 5’-CATAACTTACGGTAAATGGCCCG-3’ and CAG_rev: 5’-CGTCAATAGGGGGCGTACTTG-3’.

### Screening the random AAV display peptide library in the common marmoset in vivo

Marmoset experiments were conducted at the Neu Encepharm GmbH animal facility (Göttingen, Germany). In vivo selection of the random AAV2-peptide libraries was performed in six 2–3 years old adult male marmoset monkeys (Callithrix jacchus) between June 2015 and May 2017. At the facility of Neu Encepharm animals were kept according Guideline 2010/63 EU. The marmosets were kept pairwise in wire mesh cages (80 × 65 × 150 cm), equipped with sitting boards, sleeping boxes and branches for climbing. The animals were fed a laboratory diet (ssniff® Mar V384; ssniff® Spezialdiäten GmbH, Soest, Germany) ad libitum, a fiber and vitamin D3 enriched morning mesh (20 g/animal) and fruit and vegetables in the afternoon (30 g/animal). Water was always available. Temperature in the animal housing rooms was 26 ± 1.5 °C and humidity 60–80%. Health of all animals kept in the colony was continuously monitored and recorded. Bacteriology of fecal samples and drinking water was carried out in 3 -months intervals.

As approved by the ethics review board, *n* = 1 animal was used per selection round. Age-matched male animals were used without further randomization or blinding of the investigators, as animals were not allocated into different treatment groups for further statistical analysis. The body weight of the animals at the start of the experiment was 389 ± 78 g. On experimental days, food was withdrawn in the morning. Four hours later animals were sedated by injection of 0.5 ml/kg but not less than 0.2 ml alfaxalone per animal (10 mg/ml; Alfaxan®, Zoetis GmbH, Berlin) and a peripheral venous catheter (Vygon, O.D. 0.6 mm) was temporarily placed into the saphenous vein. Up to 6 × 1012 genomic library particles were injected in a total volume of 200 µl dissolved in Hanks’ balanced salt solution (HBSS) via the catheter. Additional information on the marmosets’ age, weight and the administered AAV doses can be found in Supplementary Table 1. Animals were kept in a small recovery cage for approximately one hour until they were fully conscious again. Thereafter they were held in an extra room together with their cage mate until the end of the experiment. After AAV-injection animals were scored at 2, 4, 6, 20, 28 and 44 h post injection for their motor activity, food intake, grooming activity, general behavior and absence of diarrhea. No impairments were observed, and all 6 animals finished the experiment. On the second day after AAV-injection food was withdrawn in the morning. After the last scoring at 44 h animals received an intramuscular injection of 1 ml/kg alfaxalone (10 mg/ml), 0.05 ml/animal glycopyrronium bromide (0.2 mg/ml) and 0.025 ml /animal diazepam (5 mg/ml). When the anesthesia was fully effective animals were sacrificed by I.v.injection of 0.3 ml Pentobarbital (300 mg/ml). Bodyweight did not change during 44 h after AAV injection and was 384 ± 76 g. Organs were harvested under sterile surgical conditions and immediately frozen on dry ice. The brains as well as various control organs were used to isolate and amplify AAV library particles and for subsequent analysis. To this end, total tissue DNA was extracted using a tissue homogenizer (Precelly’s 24 tissue homogenizer, Peqlab) and the DNeasy tissue kit (Qiagen) according to the manufacturer’s instructions. Total tissue DNA was quantified using a spectral photometer (Nanodrop ND-2000C (Peqlab). The random oligonucleotides contained in AAV library particles from the different tissues were amplified by PCR using the *cap*-specific primers 5’-ATGGCAAGCCACAAGGACGATG-3’ and 5’CGTGGAGTACTGTGTGATGAAG-3’. The PCR-amplified oligonucleotides were used to produce secondary libraries for four further selection rounds. Preselected secondary libraries were prepared like the primary naïve library (as described above).

### Next-generation sequencing of AAV peptide libraries

AAV library DNA was isolated and PCR-amplified with AAV *cap*-specific primers from marmoset brain and control tissues (see above). Amplicon libraries for NGS by the illumina MiSeq System were generated from the corresponding peptide library DNA in two subsequent PCR reactions. In a first PCR reaction one of the following primer pairs was used to attach binding sites for illumina index sequences (italic) to the *cap* fragments of the AAV peptide library (underlined):

Afw: 5’-*ACACTCTTTCCCTACACGAC*GCTCTTCCGATCTGCTCCAGAGAGGCCAGAGAG-3’,

A rev: 5’-*TGACTGGAGTTCAGACGTGT*GCTCTTCCGATCTATGAGCATCTGCGGTGGCCGCCTG-3’

Bfw: 5’-*ACACTCTTTCCCTACACGAC*GCTCTTCCGATCTTAGCTCCAGAGAGGCCAGAGAG-3’

Brev:5’-*TGACTGGAGTTCAGACGTGT*GCTCTTCCGATCTCATCATCTGCGGTGGCCGCCTG-3’

C fw: 5’-*ACACTCTTTCCCTACACGAC*GCTCTTCCGATCTATCTCTCCAGAGAGGCCAGAGAG-3’

Crev: 5’-*TGACTGGAGTTCAGACGTGT*GCTCTTCCGATCTTCATCTGCGGTGGCCGCCTG-3’

In a second PCR, the amplicons were extended by illumina index sequences and bar codes using the following primers: NGS fw: 5’-AATGATACGGCGACCACCGAGATCTACACTCTTTCCCTACACGAC-3‘

NGS rev: 5‘-CAAGCAGAAGACGGCATACGAGAT(Barcode)GTGACTGGAGTTCAGACGTGTG-3‘. DNA solutions with5 nmol/l DNA were used for sequencing.

### Bioinformatic processing of NGS data

Reads of individual samples were demultiplexed by sorting them according to their prefix, keeping only those whose prefix was identical to one of the custom index sequences incorporated during library preparation. The demultiplexed reads were searched for exact matches of the two flanking sequences GAGAGGCCAGAGAGGC and GCCCAGGCGGCCACCG. The obtained library sequences were sorted into clusters of identical sequences and subsequently different filters were applied to remove artifacts. With an observed mean Phred quality score of the samples above 32, we expected that approximately 1% of all random 21mers contain sequencing errors. To minimize such errors, we assessed the frequency of every individual sequence compared to all other sequences. If a given sequence was at least 100 times less abundant than any other sequence and the Levenshtein distance between two such sequences was 1, the less abundant sequence was excluded from further analysis. Additionally, sequences were removed if they did not match the codon scheme of the peptide-encoding random oligonucleotides. The qualified sequences were translated into peptides. During this process sequences containing ambiguous bases and stop codons were filtered out.

### Scoring of peptide variants according to their relative frequency in different tissues

Peptides were scored for their organ specificity (S scores) and their enrichment (E score) as described previously according to the equation $S=1-\frac{1}{\frac{{{\rm{Rz}}}}{{{\rm{Ry}}}}+1}$ with R_z_ as relative frequency of an individual peptide sequence in the target organ (brain) and R_y_ as its relative frequency in the analyzed off-target organ (e.g. liver).The same term was applied to the enrichment E with R_z_ representing the relative frequency in the last selection round and the R_y_ representing the relative frequency in the previous selection round. Values range from 0 (only found in off-target organ/ only found in previous selection round) to 1 (only found in target organ/ only found in last selection round). Peptide sequences with the same relative frequency in the target organ and the analyzed off-target organ have a score of 0.5.

### Production and quantification of recombinant AAV2 vectors

Recombinant AAV vectors were produced by triple-transfection of HEK 293T cells. We used ITR-less AAV 1-9 packaging plasmids, each harboring the AAV2 *rep* gene and the *cap* gene of one of the respective individual AAV serotypes. For the production of the AAV2 capsid mutants, we used the capsid-modified AAV2 plasmid pXX2-187 [36] harboring the respective peptide-encoding insertions (MRGDTPG, DWPATY, ESGHGYF [24], NRGTEWD [14]). The above-mentioned AAV packaging plasmids were co-transfected into HEK 293T cells with CAG promoter-driven luciferase reporter transfer plasmids as well as the pXX6 adenoviral helper plasmid [32] using Polyfect transfection reagent (Qiagen) according to the manufacturer’s instructions. 72 h after transfection, particles from cell culture supernatant were precipitated by 10 g PEG8000 and 5.8 g NaCl per 100 ml supernatant [34], and purified by iodixanol density gradient ultracentrifugation [35] together with the AAV particles of lysed cells after three freeze-thaw cycles. Therefore, a discontinuous iodixanol gradient was prepared by subsequently underlying the harvested library particles with 15; 25; 40; and 54% iodixanol solutions, followed by ultracentrifugation at 230,000 × *g* for 70 min. The purified library particles were aspirated from the layer containing 40% iodixanol and dialyzed against HBSS. The virus titer was determined from 1:10,000 diluted samples at genomic level by real-time PCR (see above).

### Analysis of AAV biodistribution

AAV library copy numbers were determined by qPCR in extracted total tissue DNA from the different organs with Cap-specific primers (see above) using approx. 40 ng total tissue DNA as template. To analyze the biodistribution of individual AAV variants, their relative frequencies in the different organs (determined by NGS) was multiplied with the amount of AAV library genomes recovered from these organs (determined by qPCR). AAV copy numbers were normalized to injected particles per kg bodyweight and are indicated as percentage of injected particles/kg bodyweight recovered from 1 µg total tissue DNA. Since the sequencing depth did not cover the diversity of the naïve AAV library injected into the first animals, the first screening rounds were not included in the biodistribution analysis of individual AAV variants (resulting in *n* = 3 and *n* = 1 analyzed animal for the first and second screening campaign, respectively).

### Assessment of AAV luciferase reporter expression in human primary EC

Primary human endothelial cells from different organs tested negative for HIV, HBV and HCV were purchased from ScienCell and grown in the recommended medium by the same supplier at 37 °C and 5% CO_2_: brain microvascular EC (HBMEC #1000), cardiac microvascular EC (HCMEC #6000), pulmonary microvascular EC (HPMEC #3000), renal glomerular EC (#4000), hepatic sinusoidal EC (HHSEC #5000). Human hepatoma cells (HEP-G2) tested negative for mycoplasma were purchased from the German Collection of Microorganisms and Cell Cultures GmbH (DSMZ, #AAC180) and grown in RPMI medium (Gibco) containing 10% FCS. To assess the transduction efficacy of different AAV capsids (AAV1-9 as well as AAV capsid mutants) on primary human EC, we infected cells of early passage (1–5) with luciferase reporter vectors at a MOI of 25,000 genomic vector copies per cell in a minimal volume of serum-free-medium in white 96 well plates with clear flat bottoms. Two hours after virus addition, the wells were filled up with full medium containing the recommended concentration of FCS. Transgene expression was monitored after 72 h using the luciferase assay reagent (Promega) and a luminescence plate reader (Tecan Life Sciences, Männedorf, Switzerland). Per cell type, 3–5 individual experiments were performed, each at least in technical duplicates. Pan-experimental controls were used to normalize for plate-to-plate variance.

### Statistics

Power analysis and sample size estimations were performed using the G*power 3.1.9.7 software (Heinrich Heine Universität Düsseldorf). Statistical analyses of vector distribution and gene expression experiments were performed using the Prism 9 Software (Graphpad). After analyzing data for normal distribution by the Kruskal–Wallis test, we performed one-way ANOVA and Dunnett’s multiple comparison to test for differences in vector distribution or gene expression.

## Results

### AAV library screening yields a temporary increase in particles in the brain

**Fig. 1:**
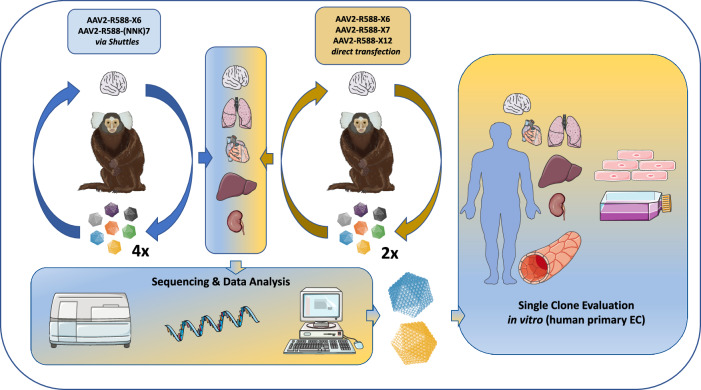
Schematic overview of the study design. Random AAV display peptide libraries either produced via intermediate AAV transfer shuttles (“two-steps”) or by direct transfection of producer cells (“one-step”) were screened in the common marmoset in vivo over four and two subsequent selection rounds, respectively. The utilized AAV libraries contained peptides either of 6, 7, or 12 amino acids in length which were encoded by the NNK scheme (7mer two-steps library) or by random trimer building blocks (all other libraries). The brain served as target organ, whereas lung, heart, liver and kidney were analyzed as controls. Enrichment of individual library clones was assessed by next generation sequencing and promising candidates were evaluated as reporter vectors in vitro on early passage primary human endothelial cells isolated from brain, heart, lung, liver and kidney. This figure contains artwork from Servier Medical Art (https://smart.servier.com/).

In a first in vivo selection approach we screened two AAV2-based capsid libraries (6mer and 7mer insertion) produced via intermediate AAV transfer shuttles (referred to as “two-steps” libraries) [9, 33] over the course of four selection rounds in the common marmoset, as a similar protocol has proven success in rodent screenings [14]. In addition, we screened three theoretically improved AAV2-based capsid libraries (6mer, 7mer, 12mer; cysteine excluded) produced by direct transfection (referred to as “one-step” libraries) [31] over the course of two subsequent selection rounds in vivo. In theory, the direct transfection protocol is expected to limit the number of wildtype-like AAV particles with affinity to heparan sulfate proteoglycan (HSPG) compared to the two-steps libraries. Assessment of biodistribution and sequencing data from a total of six selection rounds enabled us to compare the performance of the different AAV2 libraries and facilitated identification of the most promising AAV candidates, irrespective of their parental AAV display peptide libraries. Validation studies were performed on primary human microvascular EC from different organs in vitro. A schematic overview of the study design is shown in Fig. 1.

**Fig. 2:**
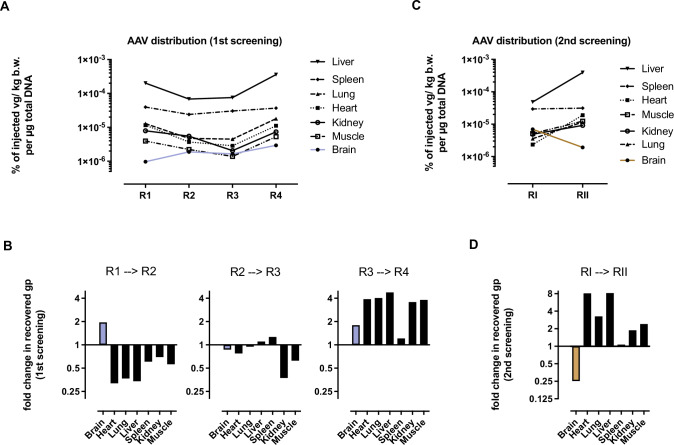
Library distribution throughout the selection process. Random AAV display peptide libraries at a dose of between 7.2 × 10^11^ genomic particles (gp)/ animal (=1.6 × 10^12^ gp/kg bodyweight) and 6 × 10^12^ gp/animal (=2.4 × 10^13^ gp/ kg bodyweight) were infused into the saphenous vein of adult male marmoset monkeys. The number of AAV library genomes recovered from different organs was quantified by qPCR 2 d after i.v. injection. Recovered AAV genomes were normalized to the number of injected particles and the animals’ body weight, indicated as the percentage of injected particles per kg bodyweight recovered from 1 µg tissue DNA. **A** AAV library biodistribution during the 1^st^screening campaign (two-steps libraries) over the course of four consecutive selection rounds. **B** AAV library biodistribution during the 2^nd^screening campaign (one-step libraries) over the course of two consecutive selection rounds. **C** Fold-change in genomic particles recovered from individual organs between consecutive selection rounds of the first screening campaign (two-steps libraries). **D** Fold-change in genomic particles recovered from individual organs between consecutive selection rounds of the second screening campaign (one-step libraries).

AAV particle distribution was determined by qPCR after each selection round to monitor the screening progress. To this end, we isolated total tissue DNA from the brain as our target organ and several peripheral organs (heart, lung, liver, spleen, kidney, skeletal muscles) and quantified the amount AAV library genomes by AAV2 *rep*-specific primers. As the injected particle dose and the animals’ body weight differed largely between the selection rounds (see Supplementary Table 1), quantified recovered library genomes were normalized to the number of injected particles per kg body weight. After the first out of four selection rounds with the two-steps libraries, most AAV library genomes could be recovered from the liver (4.8 × 10^7^vg/µg tissue DNA representing approx. 8 vg per diploid cell or 2 × 10^−4^% of injected viral genomes per kg bodyweight recovered from 1 µg tissue DNA) followed by the spleen (Fig. 2A). This was expected, given the fact that many of the AAV2 library particles still closely resembled wildtype AAV2 that shows a very similar distribution pattern. In the brain, we found 200-times less viral genomes than in the liver (2.3 × 10^5^ vg/µg tissue DNA representing approx. 0.04 vg per diploid cell or 9.7 × 10^−7%^ of injected gp/kg bodyweight recovered from 1 µg tissue DNA). We observed a strong decrease in particle numbers in the liver and all other off-target organs after the second selection round, whereas the particle number in the brain almost doubled from round 1 to round 2, indicating that the selection process was successful (Fig. 2A, B). After the third selection round, the particle number in all organs apart from liver and spleen further decreased in comparison to the round before. After the fourth selection round, however, an increase in AAV library genomes was observed for all analyzed organs including the brain (Fig. 2A, B). At this point, the number of recovered genomes from most organs reached similar values compared to the first selection round. The brain, however, showed an overall 3-fold increase in viral genome numbers from round 1–4 (Fig. 2A). The screening process was stopped at this point to avoid further enrichment of non-specific AAV variants, as indicated by the incipient increase in particle counts in off-target organs. Next, we performed two selection rounds with the hypothetically improved one-step libraries. Similar to the previous screening campaign, the biggest fraction of injected one-step library genomes was recovered from the liver after the first selection round (Fig. 2C). The number of viral genomes in the liver, however, ranged at a lower level (4.8 × 10^−5^% of injected vg/kg bodyweight recovered from 1 µg tissue DNA) than in the previous screening with the two-steps libraries which might indicate fewer HSPG-blinded library particles being included in the naïve one-step libraries. As opposed to the liver, the amount of library genomes recovered from the brain of the first selection with the one-step libraries was 7-times higher than with the two-steps libraries (6.9 × 10^−6^% injected vg/kg bodyweight recovered from 1 µg tissue DNA). Surprisingly, the second selection round with the one-step libraries led to a marked decrease in viral genomes in the brain and to a strong increase in viral genomes recovered from the off-target organs including the liver (Fig. 2C, D). We therefore discontinued the screening campaign already after the second selection round and next analyzed the change in library composition during the course of the total screening campaign.

### Marmoset in vivo screening leads to enrichment of capsid variants displaying peptides of different lengths

**Fig. 3:**
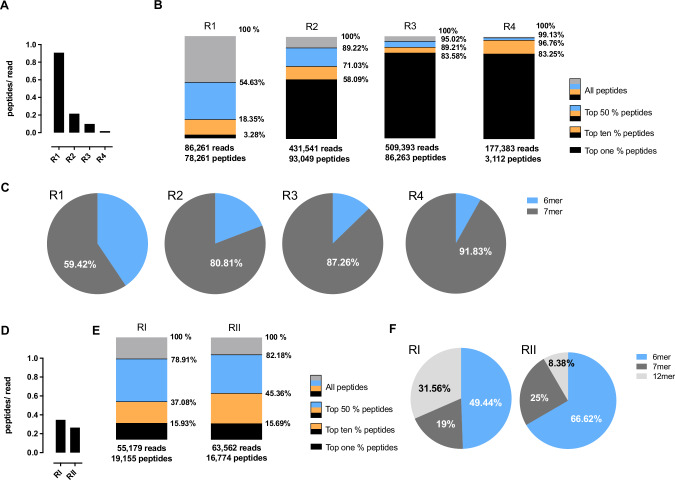
Change of library composition during the selection process. After each selection round, the recovered AAV library particles from the brain were analyzed by NGS to monitor library diversity and peptide composition. **A** Number of different peptides per sequencing read (mean) during the 1^st^ screening campaign (two-steps libraries) indicating a decrease of peptide diversity over the course of four subsequent selection rounds. **B** Bar graphs indicating the frequency of the most abundant peptides (top 1%; top 10%; top 50%) as percentage of all sequencing reads during the 1^st^ screening campaign (two-steps libraries) over the course of four subsequent selection rounds. While the most frequent peptides (top 1%) only account for 3% of all sequencing reads after the first selection round, they make up more than 83% of all sequencing reads after the fourth selection round. **C** Library composition during the 1^st^ screening campaign (two-steps libraries) in terms of peptide lengths. Naïve libraries (not shown) contained equal amounts of 6mer and 7mer peptides. **D** Number of different peptides per sequencing read (mean) during the 2^nd^ screening campaign (one-step libraries) showing a moderate decrease in peptide numbers over the course of two subsequent selection rounds. **E** Bar graphs indicating the frequency of the most abundant peptides (top 1%; top 10%; top 50%) as percentage of all sequencing reads during the 2^nd^ screening campaign (one-step libraries). While the most frequent peptides (top 1%) already accounted for about 16% of all sequencing reads after the 1^st^ selection round, this value barely changed after the 2^nd^ selection round. **F** Library composition during the 2^nd^screening campaign (one-step libraries) in terms of peptide lengths. Naïve libraries (not shown) contained equal amounts of 6mer, 7mer and 12mer peptides.

In addition to the qPCR-based quantification of library particles, the screening process was monitored by NGS. After each selection round, we sequenced 100,000–500,000 library genomes obtained from the brain and four off-target organs (heart, lung, liver, kidney). No obvious differences were detected between the different organs in terms of library diversity. After the first selection round with the two-steps libraries, almost every sequencing read from the brain corresponded to a unique peptide sequence (a mean of 0.91 peptides per read), which indicates a still quite high library diversity. During subsequent selection rounds, the peptide diversity declined markedly in favor of fewer strongly enriched peptides, indicated by a mean of 0.18 individual peptides per sequencing read after the fourth selection round (Fig. 3A). While the first percent of most frequent peptides in the brain accounted for only 3% of all sequencing reads from this organ after the first selection round (=3 times more than expected in case of equal peptide distribution), the value rapidly increased up to roughly 58% after round two, and around 83% after rounds three and four (Fig. 3B). Similarly, the upper half of most abundant peptides in the brain (top 50%) accounted for 54% of all sequencing reads from this organ after the first selection round, increasing to 89% after round two, 95% after round three, and over 99% after round four (Fig. 3B). The decreasing library diversity in favor of strongly enriched peptide sequences, was accompanied by a relative increase in particles displaying 7mer peptides. Being injected at equimolar ratio into the first animal (6mer, 50%; 7mer, 50%), the fraction of recovered 7mer-displaying particles increased from roughly 59% after the first selection round to almost 92% after selection round four (Fig. 3C). Analyzing the one-step libraries, we saw a faster reduction in library diversity already after the first selection round (indicated by 0.35 peptides per read), with moderate further reduction in diversity after a second selection round (indicated by 0.26 peptides per read) (Fig. 3D). In line with the decrease in peptide diversity, the enrichment of individual clones was strong already after a first selection round with the top percent of most frequent peptides accounting for almost 16% of all reads (Fig. 3E). As opposed to the screening with the two-steps libraries, both screening rounds with the one-step libraries were accompanied by a relative increase in capsid variants displaying 6mer peptides, on the cost mostly of 12mer peptides which only accounted for 8% of recovered clones after the second selection round (Fig. 3F).

### Different AAV libraries yield distinct peptide motifs

**Fig. 4:**
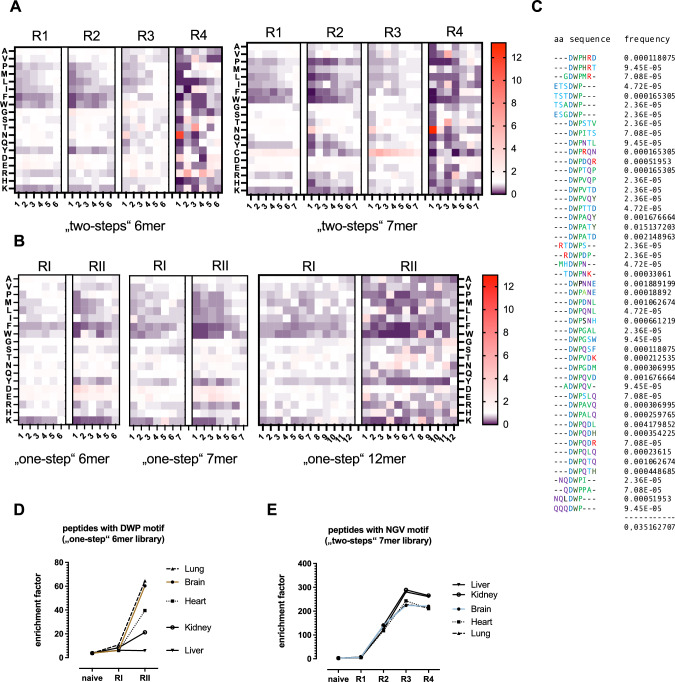
Change in amino acid distribution and peptide motifs. After each selection round, the recovered AAV library particles from the brain and peripheral organs were analyzed by NGS to monitor amino acid distribution and potential peptide motifs. **A** Change of amino acid frequencies in AAV library peptides recovered from marmoset brains during the course of the 1^st^screening campaign (4 rounds with the two-steps libraries). The fold-change in frequency of each amino acid at each position compared to the corresponding naïve AAV libraries (R0; not shown) is indicated as heatmap. Amino acid frequency either decreased (blue), did not change (white) or increased (red). **B** Change of amino acid frequencies in AAV library peptides recovered from marmoset brains during the course of the 2^nd^screening campaign (2 rounds with the one-step libraries). **C** One-step 6-mer library-derived peptide sequences recovered from marmoset brains harboring a “DWP” motif that confers relative de-targeting from the liver. The frequencies of individual DWP-harboring peptides are indicated. **D** Fold enrichment of DWP-harboring peptides in indicated organs during the course of two selection rounds with the one-step 6-mer library. **E** Fold enrichment of NGV-harboring peptides in indicated organs during the course of four selection rounds with the two-steps 7-mer library.

AAV-displayed peptides recovered from the brain and control organs were analyzed for their amino acid distribution. Enrichment of certain amino acids was observed within 6mer and 7mer peptides, irrespective of the analyzed organ. Heatmaps indicating the change in amino acid frequency during the selection process (taking into account the abundancy of each individual peptide) are shown in Fig. 4 for the brain as target organ. In early selection rounds, the frequency of the initially underrepresented aromatic amino acids phenylalanine (F) and tryptophan (W) as well as of the positively charged lysine (K) further decreased compared to the naïve two-steps libraries (Fig. 4A). Remarkably, however, selection rounds three and four led to strong enrichment of asparagine (N) at first peptide position, as well as of valine (V), tyrosine (T) and isoleucine (I) in position three, irrespective of the peptide length. After the fourth selection round with the two-steps libraries, as many as 95 out of the top 100 enriched peptides presented N as first amino acid. In addition, the frequency of the positively charged arginine (R) increased in several peptide positions (Fig. 4A; 6mer: position 1-6; 7mer: position 4). Such NXXRXXX amino acid pattern is known from many previous AAV screening campaigns, possibly stabilizing the AAV capsid or generally enhancing transduction [37] and has already been described to increase particle affinity to heparan sulfate proteoglycan in the context of AAV2 [31]. The emergence of this sequence motif might, at least partially, be explained by the production of the two-steps libraries which requires transduction of HEK 293T cells by chimeric AAV transfer shuttles in an intermediate step between every selection round. Curiously, in the 7mer two-steps library we also observed relative enrichment of cysteine (C) in screening round 1 and 3 compared to the naïve AAV library (Fig. 4A). Cysteine previously has been shown to be highly underrepresented in AAV display peptide libraries and is believed to be detrimental to the AAV capsid [29, 30]. Overall, the one-step libraries showed the same decrease of the initially underrepresented aromatic amino acids phenylalanine (F) and tryptophan (W) as well as of the positively charged lysine (K). The enrichment of N in the first peptide position as well as of V and T in peptide position three, however, was less profound than in the two-steps libraries. Instead, the two screening rounds with the one-step libraries led to an enrichment of the negatively charged residues aspartic acid (D) and glutamic acid (E) irrespective of the peptide position in case of the 6mer and 7mer libraries. The 12mer library showed a less clear pattern with multiple amino acids being more strongly over- or underrepresented at different peptide positions.

Apart from the above-mentioned dominant non-target specific amino acid patterns, we identified enrichment of another completely different motif comprising the amino acids “DWP” during our screening of the one-step 6mer library which did not become apparent in the overall amino acid distribution (Fig. 4C). About 3.5% of all 6mer library particles recovered from the brain of the second selection round accounted for DPW-harboring clones (Fig. 4C). During the screening, the DWP motif strongly enriched in different analyzed organs with exception of the liver (Fig. 4D). In the brain and the lung, the DWP motif was most prominently enriched being 60-fold more abundant than expected (expected frequency = 0.000583 considering random amino acid distribution). Slightly less profound enrichment of the DWP-motif was seen in the heart (40-fold) and the kidney (21-fold) (Fig. 4D). Interestingly, one of the five top 100 clones that were recovered from the brain of the fourth selection round with the two-steps libraries not beginning with N also displayed the DWP motif (DWPQSM). NGV, a different motif that could be identified in the two-steps libraries, on the other hand, enriched non-specifically in all organs, with the most profound enrichment in liver and kidney, where it was about 300 times more frequent than expected after four selection rounds (Fig. 4E).

### The “DWP” motif confers improved homing to marmoset brain, lung and heart and enhanced transduction of human microvascular endothelial cells

**Fig. 5:**
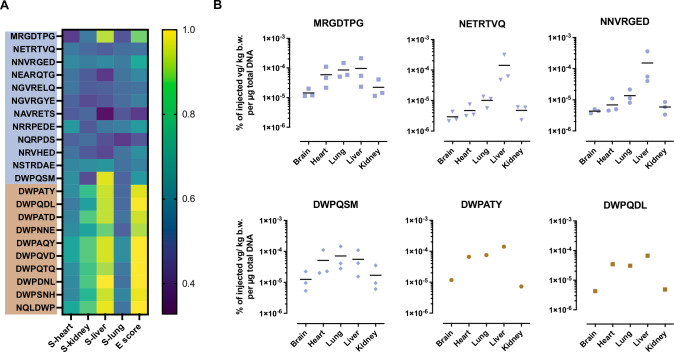
Scoring and biodistribution of individual AAV mutants in marmosets. The recovered AAV library particles from the brain and peripheral organs were analyzed by NGS after each selection round to monitor amino acid distribution and emerging peptide motifs. **A** Organ specificity scores (S) and enrichment scores (E) of selected AAV capsid mutants recovered from marmoset brains indicated as heatmap (scores ranging from 0 to 1). A high S-score indicates relative enrichment in the brain compared to the respective control organ (0 = only in control organ; 0.5 = equal frequency in brain and control organ; 1 = only in brain). A high E-score indicates relative enrichment during the screening campaign. Peptide sequences of individual AAV mutants from the first screening campaign (two-steps libraries) are indicated in the upper blue box, while AAV mutants from the second screening campaign (one-step libraries) are shown below (brown box). **B** Biodistribution of the most abundant and best-scoring capsid mutants from both screening campaigns. Individual data points represent individual animals from different selection rounds (one animal per round). The first selection rounds were excluded from analysis, since NGS did not cover the diversity of the naïve AAV libraries. Variants from 1^st^ screening campaign: screening rounds 2–4 (*n* = 3 animals), variants from 2^nd^ screening campaign: screening round 2 (*n* = 1 animal). MRGDTPG: Most frequent and best-scoring variant from 1^st^ screening campaign (two-steps 7-mer library). NETRTVQ & NNVRGED: Most frequent variants from 1^st^ screening campaign showing the non-specific NXXRXXX pattern (two-steps 7-mer library). DWPQSM: Most frequent DWP-harboring variant from the 1^st^ screening campaign (two-steps 7-mer library). DWPATY & DWPQDL: Most frequent variants from the 2^nd^ screening campaign harboring the DWP motif (one-step 6-mer library).

After analyzing the amino acid distribution of enriched AAV capsid-displayed peptides recovered from the brain (Fig. 4) and control organs, we continued our analysis by scoring individual peptide sequences according to their enrichment and relative organ specificity. To this end, we applied scores for the enrichment between selection rounds (E) and the organ specificity (e.g. S_liver_: brain vs. liver) ranging from 0 (only found in off-target organ) to 1 (only found in target organ) as previously described [24]. Scores of representative clones from the different AAV libraries are indicated in Fig. 5A. Among the AAV variants displaying the dominant amino acids N, V, T and R in the first peptide positions (the ten most frequent of which are depicted in Fig. 5A), no candidate yielded promising scores that would indicate relevant enrichment in the brain compared to the off-target organs. Scores of the most abundant peptide from the two-steps libraries (MRGDTPG) not presenting the dominant motif, however, indicated enrichment in the brain as target organ compared to the liver but not compared to the other analyzed off-target organs (Fig. 5A). The above-mentioned DWP-presenting variant DWPQSM from the two-steps libraries as well as the most frequent DWP-presenting peptides recovered from the brains of animals treated with the one-step libraries showed a similar pattern to the MRGDTPG variant with relative enrichment in the brain compared to the liver (Fig. 5A). Further, most DWP-presenting peptides showed relative enrichment in brain versus heart and kidney. None of the recovered clones showed relevant enrichment in the brain compared to the lung. Next, we analyzed the biodistribution of the most promising individual AAV variants and some of the most frequent but potentially non-specific controls during the screening procedure in marmoset monkeys by taking into account the number of recovered AAV genomes from each organ and the individual variants’ relative frequencies (Fig. 5B). Here, we saw increased homing to brain (2.5-fold), heart (10-fold) and lung (6-fold) for the MRGDTPG variant as well as for the top-enriched DWP-presenting clones DWPQSM (two-steps), DWPATY and DWPQDL (both one-step) in comparison to the most frequent AAV variants displaying the dominant amino acids N, V, T and R in the first peptide positions (NETRTVQ and NNVRGED).

**Fig. 6:**
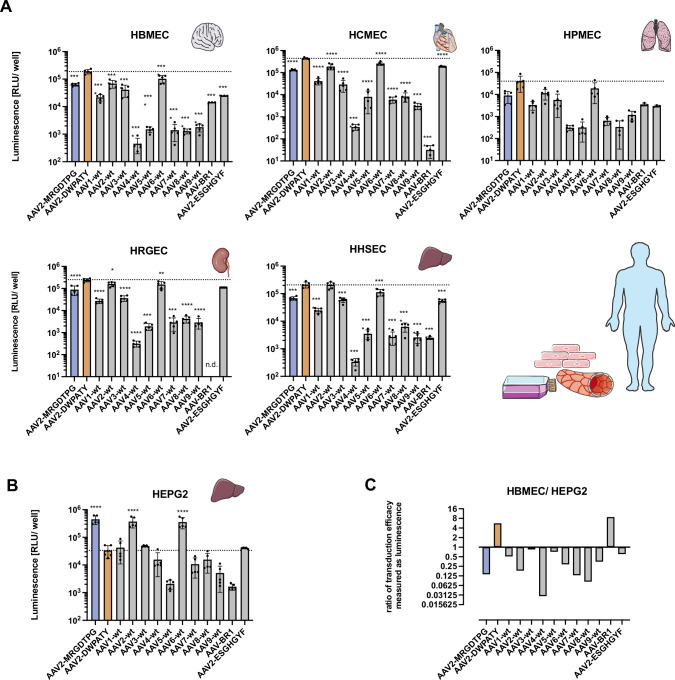
Vector-mediated transgene expression in primary human endothelial cells from different organs. Transduction efficacy of selected AAV capsid variants AAV2-MRGDTPG (blue) & AAV2-DWPATY (orange) was assessed on primary human endothelial cells from different organs. Natural AAV serotypes 1–9, and previously described capsid mutants based on AAV2 (AAV-BR1 & AAV2-ESGHGYF) were used for comparison. Luciferase reporter vectors were added to primary cells (passage 1–5) at a multiplicity of infection (MOI) of 25,000 vector copies per cell. Vector-mediated bioluminescence was measured after 48 h. Bar graphs represent the mean +/−SD with indicated individual data points; n.d. not detected. We performed *n* = 3–5 individual experiments (each at least in technical duplicates). Transduction efficacy of all variants was compared to the novel AAV2-DWPATY variant (indicated as dotted line) by one-way ANOVA, followed by Dunnett’s multiple comparison. *p < 0.05; **p < 0.01; ****p < 0.0001. **A** Vector-mediated transgene expression in human brain microvascular endothelial cells (HBMEC), human cardiac microvascular endothelial cells (HCMEC), human pulmonary microvascular endothelial cells (HPMEC), human renal glomerular endothelial cells (HRGEC) and human hepatic sinusoidal endothelial cells (HHSEC). **B** Vector-mediated transgene expression in the human hepatoma cell line (HepG2). **C** Ratio of transgene expression in HBMEC & HepG2 as indicator of EC selectivity. This figure contains artwork from Servier Medical Art (https://smart.servier.com/).

Since we observed improved homing of the MRGDTPG and the DWP variants to several major organs and since vascular ECs represent the first barrier for circulating AAV particles, we hypothesized that the novel AAV variants have an enhanced affinity for vascular ECs. We therefore compared their capacity to transduce primary human microvascular EC from different organs with multiple natural AAV serotypes, including the parental wildtype AAV2, as well as with other published brain EC-homing variants from our lab (Fig. 6).

Among the natural AAV serotypes, AAV2 and AAV6 showed the most efficient transduction of human vascular EC (Fig. 6A) which is consistent with previous in vitro studies [38]. While the AAV2-MRGDTPG variant isolated from the two-steps libraries surprisingly performed less efficient on all tested EC than parental AAV2, the analyzed DWP-harboring AAV2-DWPATY variant showed an at least doubled transduction efficiency on human brain microvascular EC (HBMEC; 2.75-fold), human cardiac microvascular EC (HCMEC; 2.3-fold) and human pulmonary microvascular EC (HPMEC; 3.3-fold) compared to wildtype AAV2. No relevant difference in the transduction efficiency between wildtype AAV2 and AAV2-DWPATY was seen on human renal glomerular EC (HRGEC) and human hepatic sinusoidal EC (HHSEC) as shown in Fig. 6A, which is consistent with the biodistribution in marmosets. The AAV-BR1 variant isolated from murine brain EC transduced HBMEC and all other tested cells at least one order of magnitude less efficiently than the novel AAV2-DWPATY variant, attesting to its species-restricted tropism. AAV2-DWPATY further transduced HPMEC and all other tested cells more efficiently than the AAV2-ESGHGYF variant isolated from murine pulmonary EC. Opposed to vascular EC, human hepatoma cells (HepG2) showed over 90% reduced transduction by the AAV2-DWPATY variant compared to wildtype AAV2, whereas the AAV2-MRGDTPG variant did not differ from wildtype AAV2 (Fig. 6B). Half of the 12 compared AAVs transduced HepG2 cells more efficiently than AAV2-DWPATY. Among the different tested AAVs, only the novel AAV capsid mutant AAV2-DWPATY and the murine brain EC-targeted variant AAV-BR1 transduced human brain microvascular EC more efficiently than HepG2 human hepatoma cells (Fig. 6C; AAV2-DWPATY: 5.6-fold expression ratio HBMEC/ HepG2). To assess whether our novel AAV variants are blinded for HSPG and the observed transduction profile is mediated by alternative receptor binding, we performed a heparin assay. While the non-specific AAV2-MRGDTPG variant showed a reduced heparin affinity by approximately 80%, AAV2-DWPATY showed a reduced heparin affinity by only 45% compared to the parental unmodified capsid (Supplementary Fig. 1). It remains open whether the observed tropism is mediated by binding to additional cell surface molecules apart from HSPG, improved intracellular processing, or by a combination of both.

## Discussion

Engineered AAV capsid libraries, either displaying random peptide insertions or comprising shuffled AAV capsid fragments from different serotypes, have successfully been screened in rodent models to identify AAV mutants with improved targeting properties [12, 14, 24, 39–42]. It has become apparent, though, that AAV mutants having been isolated from mice may not show the same tropism in primates or humans [17, 43]. Interestingly, some AAV variants that mediated improved transduction of cerebral endothelial cells in mice preferentially crossed the BBB and transduced parenchymal brain cells in non-human primates (NHP) [43]. Recently, several groups therefore conducted AAV library screenings in NHPs which prompted identification of AAV capsid mutants with improved targeting properties towards the retina [44] or the CNS [23, 45]. Being interested in vascular biology, we aimed to identify AAV variants with improved transduction of cerebral endothelial cells without conferring notable crossing of the BBB in NHPS and humans, as such variants are still lacking. Since the serotype AAV2 has been shown to transduce different human (endothelial) cells more efficiently than AAV9 (at least in vitro) [38], here we screened AAV2-displayed peptide libraries in NHPs in vivo, instead of the frequently used libraries based on AAV9 that generally confer stronger CNS tropism. As screening model, we chose the common marmoset, a monkey that does not require administration of high doses of AAV library particles due to its small size. The common marmoset, belonging to the New World monkeys (*Platyrrhini*), and humans, belonging to the Old World monkeys (*Catarrhini*), diverged from common ancestors ~40 million years ago, whereas the ancestors of rodents and primates split apart more than 80 million years ago. Marmosets, therefore, resemble humans much more closely than mice and the common marmoset is considered an adequate model for brain research [46, 47]. We chose the same AAV library backbone with random peptide insertion at amino acid position R588 of the AAV2 cap gene that had already been used very successfully in rodent screenings, yielding the brain EC-targeted AAV-BR1 variant [14]. We therefore also stuck to the same screening protocol, used the same application method (i.v.) and did not change the incubation time (two days), although this is comparably short and might not be sufficient to complete intracellular capsid trafficking and stable episome formation. However, to further increase the likelihood of identifying useful variants, we compared different AAV library production methods that are expected to interfere differently with the screening process [31], namely the direct transfection of producer cells (“one-step”) and the transduction of producer cells by intermediate library transfer shuttles (“two-steps”) [9, 33] which we had used in some of our previous screenings [14, 24, 28]. In addition, we used peptide insertions of different length (6mer, 7mer, 12mer) to enhance library diversity.

The PCR-based amplification of AAV library DNA recovered from target tissue, as applied in this study, is a simple screening method that has been used successfully by us and others in different settings. While this method in theory only requires capsid attachment to the cell, the Cre-recombination-based AAV targeted evolution (CREATE) [12, 25], a refined DNA-based AAV library approach, ensures successful AAV internalization and intracellular processing that eventually enables recombination of uncoated library DNA. The CREATE library, however, requires transgenic animals, which limits its application to rodents. In principle, RNA-based directed evolution approaches show several benefits over the DNA-based methods as they ensure all relevant steps of successful transduction (cellular uptake, intracellular processing, and gene expression). Some of the more recent directed evolution approaches, therefore, either harness the native AAV p40 promoter [48] or cell type-specific promoters [49] to ensure successful transcription of AAV library DNA.

It has already been shown that random AAV display peptide libraries contain a bulk of undesired capsid mutants that closely resemble the unmodified wildtype AAV2 capsid with strong affinity to heparan sulfate proteoglycan (HSPG), potentially rendering particle homing to the liver [30, 31]. Further, the increased transduction of pre-defined target tissue by many peptide-displaying AAV variants identified so far is accompanied by a still substantial transduction of liver tissue, indicating incomplete abrogation of natural AAV receptor interactions [26, 28, 40]. This also holds true for most brain-targeted AAV variants derived from AAV library in vivo screenings [12, 13, 15, 25]. In theory, during the in vivo selection, the majority of non-specific capsid mutants should decrease in favor of potentially very few variants being more selective for the target cells. In light of this idea, the steady increase in particle numbers in the brain as target organ during our screening of the two-steps libraries was expected, despite the fact that most library particles were still recovered from the liver and other peripheral organs. The strong decrease of library particles in the brain and the enrichment of apparently non-specific variants in the off-target organs observed during the screening of the one-step libraries, however, lack a definite explanation. A potential advantage of certain non-specific particles during the preparation of the preselected secondary particle libraries, as well as superior capsid stability, antibody escape, or intracellular processing, might have contributed to the observed phenomenon.

Like most so-far reported brain-targeted AAV library mutants [12, 13, 15, 25], the best-scoring variants from our screening still show substantial homing to multiple peripheral organs. Interestingly, during our NHP screening with the two-steps libraries we enriched the same potentially non-specific amino acid pattern that had already been shown to be fostered by the use of the intermediate library transfer shuttles during the production process [31]. The enriched peptide motif NXXRXXX does not only enhance non-specific transduction by AAV2 but has also been reported for other AAV serotypes such as AAV9, possibly due to optimized intracellular processing of AAV particles harboring such peptides [37]. If this is true, AAV mutants displaying the NXXRXXX motif could enrich during in vivo screenings without organ specificity. Even one of the most recently described AAV variants with increased CNS targeting properties, the brain-homing variant AAV-X1 (GNNTRSV), presents a very similar amino acid pattern shifted by only one position. Being isolated from NHPs by screening an AAV9 library in vivo, the XNXXRXX-harboring AAV-X1 variant still confers strong tropism for liver tissue, suggesting an overall increased fitness of NXXRXXX- or XNXXRXX-harboring peptides rather than specific receptor targeting. The introduction of additional point mutations into the AAV library backbone might help to prevent natural receptor binding in the future. Indeed, introducing an R585A mutation into the AAV2 cap gene has been shown to efficiently prevent HSPG binding and transduction of peripheral organs in vivo [50], whereas the W503A mutation in the AAV9 cap gene is sufficient to prevent binding to galactose [51].

Although marmosets previously were shown to be seropositive for AAV9 and AAVrh.10, they seem to have lower neutralizing antibody titers than other NHPs such as chimpanzee, cynomolgus macaque, and rhesus macaque [52]. Since the used marmosets were housed in a clean laboratory environment, we did not expect neutralizing antibodies that would interfere with the in vivo selection, although an antibody screening might have been beneficial for the interpretation of our data. Differences in recovered library genomes between selection rounds, for example, or the enrichment of cysteine observed in the two-steps 7mer library after the third selection round might be related to different neutralizing antibody titers. Variances in AAV production quality might further have contributed to the different numbers of recovered AAV library genomes between consecutive selection rounds.

Using a previously published scoring system [24], we believe to have identified the most promising AAV capsid variants enriched during our in vivo screening, although AI-assisted or other novel bioinformatic tools might help to identify additional suitable variants for the brain and other analyzed organs [53, 54]. The new DWP motif-presenting variants show enhanced homing to brain tissue in marmosets compared to the most strongly enriched non-specific NXXRXXX variant. Further, the DWP motif-presenting DWPATY variant showed enhanced transduction of HBMEC compared to the tested natural AAV serotypes (AAV1-9) and previous AAV2 capsid mutants from our lab. The mechanisms behind the observed enhanced homing to the marmoset brain and increased transduction of human vascular EC, so far, have not been investigated in detail. Although the DWP motif confers a net negative charge to the inserted peptide, which commonly ablates HSPG binding [30], AAV2-DWPATY only shows a reduced heparin affinity by about 45% as compared to the unmodified parental serotype AAV2. At this stage, we cannot rule out that AAV2-DWPATY, in addition to HSPG, shows affinity to other cell surface molecules that are not bound by wildtype AAV2. Vascular endothelial cells are rich in membrane proteins and transporters that might serve as additional entry factors [55]. Improved intracellular processing, as seen for other AAV capsid mutants [37], might be an alternative explanation for the observed transduction profile. It is conceivable, for example, that peptide insertion might influence the pH-dependent conformational shift of the AAV capsid, fostering endosomal membrane disruption and virion escape via the exposure of the phospholipase A2 domain [56]. Interestingly, the ideal peptide length cannot be concluded from our screening. While the most successful peptide from the screening of the two-steps libraries in terms of enrichment and specificity was a 7-mer (MRGTDPG), the screening of the one-step libraries yielded more successful 6-mer peptides (the DWP-presenting ones). Therefore, it might be useful not only to rely on multiple AAV serotypes but also peptides of different lengths in future AAV library screenings in order to enhance library diversity.

Although we have not isolated AAV variants selectively targeting brain EC, we identified a DWP peptide motif potentially targeting (microvascular) EC more broadly. The enrichment of the vascular-targeted DWP motif in the one-step but not in the two-steps libraries confirms the detrimental effect of the intermediate AAV transfer shuttles during library production that had already been hypothesized [31]. EC tropism of the identified AAV variants has not yet been validated in the marmoset. Instead, we used low-passage primary microvascular EC from different human organs to confirm endothelial tropism. At least in the in vitro setting, the DWP-presenting AAV variants outperformed the natural serotypes AAV1-9. The reduced transduction of human hepatoma cells in combination with the increased transduction of vascular EC from brain, lung, heart and kidney might render DWP-presenting AAV variants useful candidates for gene therapy of a broad range of vasculopathies. The benefit of AAV2-DWPATY over the natural AAV serotypes was most profound in human pulmonary and brain microvascular endothelial cells. Therefore, it might be an especially promising tool for pulmonary and neurovascular diseases. It remains unclear, however, how far the in vitro results are able to predict the in vivo situation in humans. Further studies need to be conducted to validate the therapeutic potential of our DWP-presenting AAV variants before moving them towards clinical application.

## Supplementary information

Supplementary Material

## Data Availability

Sequence data reported in this publication has been submitted to the European Nucleotide Archive (ENA). Data is publicly available under accession PRJEB86522. Additional data will be shared by the corresponding author upon reasonable request.
