# Correction: Identification of AAV variants with improved transduction of human vascular endothelial cells by screening AAV capsid libraries in non-human primates

**DOI:** 10.1038/s41434-025-00565-2

**Published:** 2025-09-05

**Authors:** Maria Stamataki, Julia Lüschow, Christina Schlumbohm, Malik Alawi, Lars Lunding, Eberhard Fuchs, Martin Trepel, Markus Schwaninger, Jakob Körbelin

**Affiliations:** 1 ENDomics Lab, Department of Oncology, Hematology and Bone Marrow Transplantation, University Medical Center Hamburg-Eppendorf, Hamburg, Germany; 2 German Primate Center – Leibniz-Institute for Primatology, Göttingen, Germany; 3 Bioinformatics Core, University Medical Center Hamburg-Eppendorf, Hamburg, Germany; 4 Division of Lung Immunology, Research Center Borstel, Borstel, Germany; 5 Department of Hematology and Medical Oncology, Augsburg University Hospital and Medical Faculty, Augsburg, Germany; 6 Bavarian Cancer Research Center (BZKF), Augsburg, Germany; 7 Comprehensive Cancer Center Alliance WERA, Augsburg, Germany; 8 Institute for Experimental and Clinical Pharmacology and Toxicology, University of Lübeck, Lübeck, Germany; 9 German Research Centre for Cardiovascular Research (DZHK), partner site Hamburg/Lübeck/Kiel, Lübeck, Germany

**Keywords:** Genetic vectors, Genetic vectors

Correction to: *Gene Therapy* 10.1038/s41434-025-00563-4, published online 22 August 2025

Some numbers in figure 2’s captions have to be superscripted. The previous was:

“Random AAV display peptide libraries at a dose of between 7.2 × 1011 genomic particles (gp)/ animal (=1.6 × 1012 gp/kg bodyweight) and 6 × 1012 gp/animal (=2.4 × 1013 gp/ kg bodyweight) were infused into the saphenous vein of adult male marmoset monkeys.”

The correct is:

“Random AAV display peptide libraries at a dose of between 7.2 × 10^11^ genomic particles (gp)/ animal (=1.6 × 10^12^ gp/kg bodyweight) and 6 × 10^12^ gp/animal (=2.4 × 10^13^ gp/ kg bodyweight) were infused into the saphenous vein of adult male marmoset monkeys.”

The original article has been corrected.

