# Supplementary material for: Identification of AAV variants with improved transduction of human vascular endothelial cells by screening AAV capsid libraries in non-human primates
Source: Gene Ther. 2025 Aug 22;32(5):529–41. doi: 10.1038/s41434-025-00563-4 (PMC12518125; doi:10.1038/s41434-025-00563-4)
Supplement: Supplementary file 1 — Supplementary Material [file 41434_2025_563_MOESM1_ESM.docx]

**Supplementary Information**

**Identification of AAV variants with improved transduction of human vascular endothelial cells by screening AAV capsid libraries in non-human primates**

Maria Stamataki, Julia Lüschow, Christina Schlumbohm, Malik Alawi, Lars Lunding, Eberhard Fuchs, Martin Trepel, Markus Schwaninger, Jakob Körbelin

**List of Content:**

- **Supplementary Figure 1**
- **Supplementary Table 1**

**Supplementary Figure 1: Heparin Assay.** Recombinant AAV Luciferase vectors based on different AAV2 capsids were applied to columns containing 1 ml heparin agarose (Sigma Aldrich # H6508) in a total volume of 5 ml PBS (approx. 1x10^10^ particles/ column). The columns were washed twice with 5 ml PBS and bound particles were eluted twice with 5 ml PBS containing 2M NaCl. The number of AAV particles in each fraction (flow through, wash 1 & 2, elution 1 & 2) was determined by AAV2 capsid ELISA (Progen: #PRATV; Heidelberg, Germany). The percentage of bound particles (elution 1 & 2) is indicated. We performed n = 4 individual experiments with each AAV capsid variant. Statistics: One-way ANOVA followed by Turkey’s multiple comparison test, **** = p < 0.0001.

**Supplementary Table 1. Information on animals used for the screening**

| Screening I (“two-steps libraries”) | |
| --- | --- |
| Round 1 |  |
| Animal # | #E563 |
| Sex | male |
| Date of birth | 12.03.2013 |
| Date of euthanasia | 25.06.2015 |
| Weight (injection date): | 253 g |
| Injected particles | 6x10^12^ vg total / 2.37x10^13^ vg/kg |
| Recovered AAV genomes | 3x10^6^ vg used for NGS |
|  |  |
| Round 2 |  |
| Animal # | #583 |
| Sex | male |
| Date of birth | 04.07.2013 |
| Date of euthanasia | 07.09.2015 |
| Weight (injection date): | 441 g |
| Injected particles | 2.4x10^12^ vg total / 5.44x10^12^ vg/kg |
| Recovered AAV genomes | 3x10^6^ vg used for NGS |
|  |  |
| Round 3 |  |
| Animal # | #562 |
| Sex | Male |
| Date of birth | 12.03.2013 |
| Date of euthanasia | 30.10.2015 |
| Weight (injection date): | 315 g |
| Injected particles | 3x10^12^ vg total / 9.52x10^12^ vg/kg |
| Recovered AAV genomes | 3x10^6^ vg used for NGS |
|  |  |
| Round 4 |  |
| Animal # | #E559 |
| Sex | male |
| Date of birth | 10.03.2013 |
| Date of euthanasia | 02.03.2016 |
| Weight (injection date): | 414 g |
| Injected particles | 3x10^12^ vg total / 7.25x10^12^ vg/kg |
| Recovered AAV genomes | 3x10^6^ vg used for NGS |

| Screening II (“one-step libraries”) | |
| --- | --- |
| Round 1 |  |
| Animal # | #E564 |
| Sex | male |
| Date of birth | 13.03.2013 |
| Date of euthanasia | 17.08.2016 |
| Weight (injection date): | 445 g |
| Injected particles | 7.2x10^11^ vg total / 1.62x10^12^ vg/kg |
| Recovered AAV genomes | 3x10^6^ vg used for NGS |
|  |  |
| Round 2 |  |
| Animal # | #E597 |
| Sex | male |
| Date of birth | 16.01.2014 |
| Date of euthanasia | 10.05.2017 |
| Weight (injection date): | 450 g |
| Injected particles | 2.0x10^12^ vg total / 4.44x10^12^ vg/kg |
| Recovered AAV genomes | 3x10^6^ vg used for NGS |
